# Barriers to and enablers of the HIV services continuum among gay and bisexual men worldwide: Findings from the Global Men’s Health and Rights Study

**DOI:** 10.1371/journal.pone.0281578

**Published:** 2023-05-04

**Authors:** Sonya Arreola, Glenn-Milo Santos, Diego Solares, Johnny Tohme, George Ayala

**Affiliations:** 1 Division of Prevention Science, University of California, San Francisco, San Francisco, California, United States of America; 2 Arreola Research, San Francisco, California, United States of America; 3 Center for Public Health Research, San Francisco Department of Public Health, San Francisco, California, United States of America; 4 School of Nursing, University of California San Francisco, San Francisco, California, United States of America; 5 Consultant for MPact Global Action, Auckland, New Zealand; 6 MPact, Oakland, California, United States of America; 7 Alameda County Department of Public Health, Oakland, California, United States of America; New York Blood Center, UNITED STATES

## Abstract

**Objectives:**

To assess ecological, structural, community and individual level correlates of health services utilization along a continuum of HIV care, and sexual health and support services among gay and bisexual men worldwide.

**Methods:**

Using a nonprobability internet sample of 6,135 gay and bisexual men, we assessed correlates of utilization of health services. Chi-Square Tests of Independence were performed to assess drop off along a continuum of HIV care. Multivariable logistic regression analyses using generalized estimating equation models were conducted adjusting for geographic region and clustering by country. In multivariable analyses, we determined the association between utilization outcomes, and ecologic, structural, community and individual correlates by fitting separate generalized estimating equation (GEE) logistic regression models for each of the outcomes, fitted with robust SEs, and accounting for clustering by country. Stratified by sexual identity, analyses adjusted for variables that could influence HIV-related health outcomes including racial/ethnic minority status, participant age, insurance, ability to make ends meet, as well as country-level income (income of country of residence defined by the World Bank).

**Results:**

Among men living with HIV (n = 1001), being in HIV care (n = 867) was associated with being on ART (*X*^2^ = 191.17, *p* < .001), and viral load suppression (*X*^2^ = 14.03, *p* < .001); and using ART (n = 840) was associated viral load suppression (*X*^2^ = 21.66, *p* < .001). Overall, the pattern of utilization outcomes were similar for both gay and bisexual men. For example, utilization of PrEP, being in HIV care and utilization of most of the sexual health and support services were negatively associated with sexual stigma. Whereas, utilization of most HIV prevention, and sexual health and support services were positively associated with provider discrimination. Utilization of all HIV prevention and all sexual health services were positively associated with greater community engagement, and receiving services from LGBT-led organizations. Bisexual men had higher odds of reporting provider discrimination when utilizing condom services (gay: AOR = 1.14, [0.95–1.36]; bisexual: 1.58, [1.10–2.28]), PrEP (gay: AOR = 1.06, [0.77–1.45]; bisexual: AOR = 2.14, [1.18–3.89], mental health services (gay: AOR = 1.03, [0.86–1.23]; bisexual: AOR = 1.32, [1.07–1.64]), and community-based support (gay: AOR = 1.23, [1.05–1.45]; bisexual: AOR = 1.49, [1.14–1.93]) than gay men. Bisexual men also reported higher odds of accessing services from LGBT-led organizations when utilizing PrEP (gay: AOR = 5.26, [2.50–11.05]; bisexual: AOR = 7.12, [3.16–16.04]), and community-based support/self-help groups/individual counseling (gay: AOR = 2.63, [1.72–4.01]; bisexual: AOR = 3.35, [2.30–4.88].

**Conclusions:**

It is essential that barriers to health services utilization be addressed at structural and community levels. Structural interventions should be designed to reduce sexual stigma, as well as train and sensitize healthcare providers; and strengthen community level interventions that bring gay and bisexual men together to lead comprehensive health services.

## Introduction

Gay men, bisexual men and other men who have sex with men (hereafter referred to as gay and bisexual men) [1] are 26 times more likely to acquire HIV than other adult men. In 2019 gay and bisexual men represented nearly 1 in 4 new HIV infections worldwide [2, 3]. In many countries, gay and bisexual men are still treated as criminals and denied access to the health and HIV services they need; at least 69 countries have laws that criminalize same-sex sexual relations [2, 4]. Criminalization of sex between men emboldens structural violence (social structures that put people in harm’s way) [5], including human rights abuses, violence, stigma and discrimination, all of which worsen health disparities for gay and bisexual men and their communities [6–9].

Ecological factors (environmental exposures measured through an external data source), such as criminalization of sex between men, have been found to impede access to HIV services [8, 10–12]. Structural factors such as sexual stigma and discrimination on the part of health providers; and individual level factors such as psychological distress have also been shown to obstruct access to health services including those along the HIV prevention and HIV care continuum [8–10]. Conversely, community level factors, such as community engagement, enable services access among gay and bisexual men [11, 13–15].

The persistence of barriers to HIV services is particularly concerning given that access to and utilization of HIV services are essential for achieving HIV epidemic control and prevention. For example, a 2020 study on estimates of HIV infections averted among gay and bisexual men, indicated that condom use and antiretroviral treatment averted 19% (95% uncertainty interval: 14%-25%) and 23% (15%-31%) of HIV infections that would have occurred since 1984 and 1996, respectively [16]. Although the roles of structural violence and community engagement in accessing HIV services has been examined, less is known about how these factors impact utilization of HIV services and related health needs. Therefore, research characterizing gaps along the HIV prevention and care continuum and factors that impede or enable utilization of these health services remain of high public health importance.

This study aimed to explore ecological, structural, community and individual level indicators of utilization of services along the HIV prevention and care continuum, as well as additional sexual health and support services, among gay and bisexual men worldwide. Based on previous findings on correlates of access to HIV services [8–10] and using a social-ecological framework [17, 18], we hypothesized that for gay and bisexual men in our sample: 1) living in a country that criminalizes sex between men (ecological); sexual stigma (aka, homophobia), and experiences of provider stigmatization (structural) would be negatively associated with utilization of services; and 2) engagement with gay community (community); and well-being (individual) would be positively associated with utilization of services. Finally, we hypothesized that 3) receiving care through lesbian, gay, bisexual, transgender (LGBT)-led organizations would be positively associated with service utilization and positive health outcomes.

## Methods

### Study design

This cross-sectional study was based on the fourth Global Men’s Health and Rights Survey (GMHR-4) that was launched on 4 September 2019 and remained open through to 31 March 2020 [8, 13]. Consistent with previous GMHR survey characteristics [10, 14, 19], GMHR-4 data were collected from a nonprobability internet sample of gay and bisexual men, recruited via organizational outreach, email listservs, gay dating apps, and websites. Recruitment materials were also disseminated on Hornet’s app in Latin America and Caribbean, accounting for their overrepresentation in the sample. Hornet is a free, smart-phone based “Gay Social Networking” app with over 25 million users worldwide that has been used as a means for conducting research with gay, bisexual and other men who have sex with men worldwide [20–22]. Participants were invited to complete a 20 to 30-minute online survey. After reading a consent script describing the study, only participants who selected “Yes” to “Do you wish to participate in this survey?” were able to begin answering survey questions. Eligible participants, needed to identify as male (cisgender or transgender), have had sex with another man in the last 6 months, be 18 years or older, and be able to complete the online survey in Arabic, Chinese, English, French, Indonesian, Portuguese, Russian, Spanish, Swahili or Vietnamese. No geographical restrictions were applied. Ethical approval was obtained from the Western Institutional Review Board, which determined that GMHR-4 was exempt under Category 4.

### Measures

Demographic characteristics included individual questions on country of residence, age, ability to meet one’s basic financial needs, education, relationship status, urban/rural living location, racial/ethnic background, health insurance coverage, and HIV status.

Criminalization of sex between men (an ecological factor of an environmental exposure measured through an external data source) was based on The International Lesbian, Gay, Bisexual, Trans and Intersex Association (ILGA) 2019 Report’s indication of country status of criminalization [4]: we used ILGA’s categorization of countries that still criminalize consensual same-sex sexual acts between adults. These included countries that have legal barriers to freedom of expression for gay and bisexual men in the forms of laws that explicitly ban sex between men; laws that ban self-expression or public gatherings (freedom of assembly); and others that use vague terms such as “acts against nature”, “indecency”, or “immoral acts”, that are open to arbitrary interpretation, leading to discretionary use of these norms to persecute LGBT people. If the country had criminalizing policies based on the ILGA report categorizations, the country was coded as yes for being a criminalizing country. Cronbach’s Alpha values showed good reliability for all scales used in this study.

Sexual stigma (a = 0.82) consisted of 7 item scale to measure attitudes about gay and bisexual men, *e*.*g*., *“In your country*, *how many people believe that male homosexuals are disgusting*?” with five-point Likert responses ranging from *“none”* to *“all”*.

Provider-discrimination (a = 0.87) consisted of five items measuring experiences of being discriminated against by health providers, *e*.*g*., *“In the last 6 months*, *has a health care provider refused to treat you because you are gay | bisexual | men who have sex with men (MSM)*?*”*, with five-point Likert responses ranging from *“No*, *never”* to *“Yes more than 5 times”*.

Community engagement (a = 0.72) consisted of ten items measuring level of engagement in social activities with other MSM, e.g., “*During the past 6 months*, *how often have you participated in gay | bisexual | MSM social groups or in activities such as a book or cooking club*?*”*, with five-point Likert responses ranging from “*Never”* to “*More than 12 times”*.

LGBT-led services consisted of questions asking where men received respective healthcare services, i.e., “*Where did you access this service*?” with 7 possible locations to choose from such as “*Private clinic/hospital*”. This variable was categorized as LGBT-led services if a participant selected “*Gay/MSM or LGBT focused CBO / NGO”* for at least one of the healthcare services.

Psychological wellbeing (a = .85) consisted of six items from the Psychological Domain of the World Health Organization Quality of Life (WHOQoL-BREF) questionnaire, measuring perceived sense of psychological well-being during the “*last four weeks*”, with five-point Likert responses ranging from “Never” to “Always”.

Among men living with HIV, ever having had a viral load test was assessed; for those on ART, virologic suppression was coded for those whose “current viral load” was “less than 200 copies/mL”; and CD4 count >200 was coded for those who selected “200–350” or greater.

The primary outcomes in this study were utilization of HIV prevention (condoms, HIV testing and PrEP); HIV care (HIV care and ART); and support health services (STI testing, STI treatment, mental health care, and community based counseling) over the prior six months (exceptions described below) using questions such as: ‘‘*In the last 6 months*, *how frequently have you obtained condom-compatible lubricants*?”, with five-point Likert responses ranging from “*never*” to “*more than 10 times*”. For current analysis we dichotomized the continuous outcome as “utilized” for anything equal to or more than “*Once or twice”*.

The HIV continuum presented in this report builds an HIV prevention, care and treatment services continuum using GMHR-4 survey data and an intervention and a client-centric perspective of services utilization [23]. The prevention end of the constructed continuum includes all study participants, beginning with the number of men who reported obtaining condoms in the last six months. The HIV care section includes only men living with HIV, beginning with the number of men who reported having used HIV care in the last six months. Finally, the HIV treatment end of the continuum includes men living with HIV who are on ART, beginning with the number of men who reported ever having had a viral load test.

Pre-exposure prophylaxis (PrEP) use among those who did not self-identify as living with HIV was assessed for lifetime use with the following question: ‘‘*Have you ever taken HIV medications before potentially being exposed to HIV*, *because you thought it would reduce your chances of getting HIV*?” Participants were considered to have used PrEP if they responded ‘‘yes” to this question.

Among participants living with HIV, retention in HIV care was coded if they reported one or more visits in the prior 6 months.

### Statistical analyses

Chi-Square Tests of Independence were performed to assess the relationship between being in HIV care and HIV treatment outcomes (ART, viral load testing, viral load suppression and CD4 count); and between being on ART, and viral suppression and CD4 count. We determined associations between the dichotomized outcome measures for utilization of health services described above, and the ecological, structural, community and individual level correlates using bivariable logistic regression models, using a cut-off of α<0.05 for statistical significance. Multivariable analyses, were conducted to determine the association between dichotomized outcome measures for utilization of health services and ecological, structural, community and individual level correlates, by fitting separate generalized estimating equation (GEE) logistic regression models for each of the outcomes. GEE models were fitted with robust SEs and an exchangeable correlation structure, accounting for clustering by country. Stratified by sexual identity, analyses adjusted for variables that could influence HIV-related health outcomes including racial/ethnic minority status, participant age, insurance, ability to make ends meet, as well as country-level income (income of country of residence defined by the World Bank) [10, 24, 25]. Analyses were conducted using Stata version 15.1 (StataCorp, College Station, TX).

## Results

A total of 6,135 gay and bisexual men completed the GMHR survey. Slightly more than a third of study participants completed the survey in Portuguese (35%), followed by Spanish (19%), English (15%), Arabic (9%), Russian (9%), French (6%), Vietnamese (5%), Indonesian (2%), Chinese (1%) and Kiswahili (0%). A total of 123 countries were represented in the sample. Regionally, the majority of men were from Latin America and Caribbean (55%); followed by Europe and Central Asia, Middle East and North Africa and Sub-Saharan Africa (12% each); East Asia & Pacific (8%); North America (2%); and South Asia 0.1%) (see Table 1). Twenty-two percent of respondents were from countries that criminalize sex between men. Most men (n = 5,212) were between 19 and 50 years of age and had high levels of education. More than half (n = 4,635) reported post-secondary education or higher; but fewer than half (n = 2,206) reported an ability to mostly or completely meet their financial needs. Most men (73%) reported not being in relationship, and about a quarter were from a small town or rural area. Finally, 20% were living with HIV and 0.6% reported not knowing their HIV status.

**Table 1:** Participant characteristics (N = 6,156).

Region	n	%
Latin America & Caribbean	3,379	54.9%
Europe & Central Asia	708	11.5%
Middle East & North Africa	732	11.9%
Sub-Saharan Africa	714	11.6%
East Asia & Pacific	516	8.4%
North America	104	1.7%
South Asia	7	0.1%
**Lives in Criminalizing Country**		
No	4,319	70.4%
Yes	1,816	29.6%
**Age**		
14–19	536	8.7%
20–29	2,687	43.5%
30–49	2,528	40.9%
50+	433	7.0%
**Financial Needs Met**		
Not at all	980	15.8%
A little	1,287	20.8%
Moderately	1,714	27.7%
Mostly	1,488	24.1%
Completely	718	11.6%
**Education**		
No formal education	27	0.4%
Elementary School / Primary School	68	1.1%
High School / Secondary School	1,072	17.3%
Apprenticeship / Trade worker training	385	6.2%
Postsecondary Education / College/University	3,520	56.9%
Postgraduate / Masters / Doctoral education	1,115	18.0%
**Relationship status**		
In a relationship	1,673	27.0%
Not in a relationship	4,514	73.0%
**Urban/Rural**		
a capital city	2,597	42.0%
a large city	1,900	30.7%
a suburb near a large city	547	8.8%
a small city or town	860	13.9%
a rural area or village	283	4.6%
**Racial and Ethnic Background**		
Not a racial or ethnic minority	4,731	76.5%
Racial or ethnic minority	1,456	23.5%
**Health insurance**		
I have no health care coverage	1,907	30.8%
Government-provided	1,746	28.2%
Employer-based insurance	1,165	18.8%
Private insurance through self	773	12.5%
Private insurance through a family member	368	5.9%
University Insurance	228	3.7%
**HIV status**		
Not living with HIV	4,051	79.7%
Living with HIV	1,001	19.7%
Do not know	31	0.6%

### HIV prevention, care and treatment continuum

**Fig 1:**
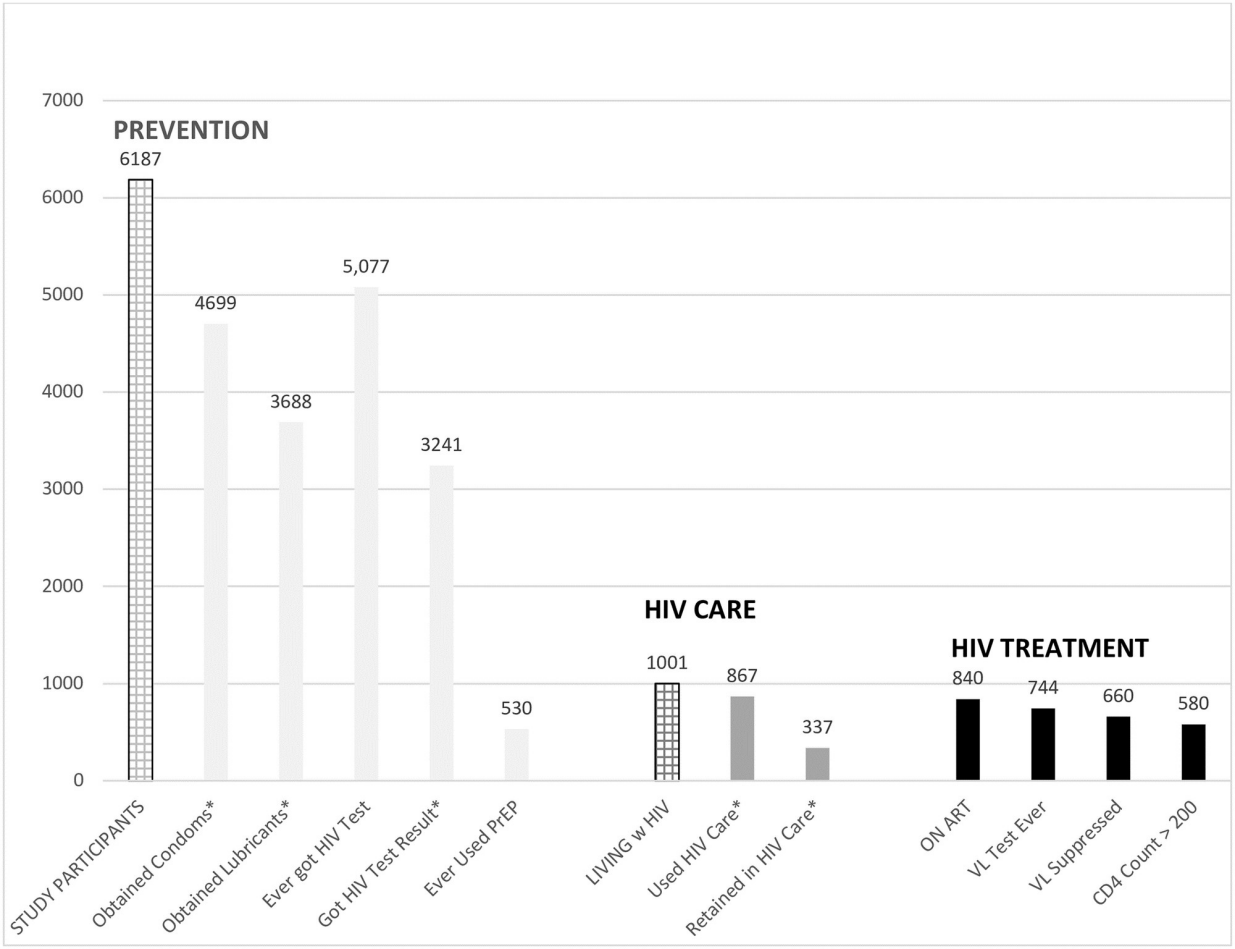
HIV prevention, care and treatment continuum among gay and bisexual men from the GMHR-4 Study. *Over last six months. The prevention end of the continuum includes all study participants (n = 6,187); the HIV care and treatment ends are specific to participants living with HIV (n = 1,001); service utilization steps were reported over the prior 6 months unless otherwise specified.

We constructed an HIV services continuum using utilization data from GMHR 4.0 (Fig 1). Among all participants (N = 6,187), 76% obtained condoms; 60% obtained lubricants, 52% received their HIV test results; and 27% used HIV prevention programs in prior 6 months. Eighty-two percent of participants reported ever getting HIV tested and 9% reported ever using PrEP. On the HIV care and treatment end of the services continuum (N = 1001), 87% of men living with HIV reported using HIV care, 34% reported retention in HIV care; and 84% reported being on ART. Among men on ART (N = 840), 74% reported ever having had a viral load test; 66% reported being virologically suppressed; and 58% reported having a CD4 count greater than 200.

Among men living with HIV, being in HIV care was significantly associated with being on ART (*X*^2^ = 191.17, *p* < .001) and viral load suppression (*X*^2^ = 14.03, *p* < .001). Among men living with HIV, being on ART was significantly associated viral load suppression (*X*^2^ = 21.66, *p* < .001).

### Utilization

Odds ratios for correlates of health services utilization are reported below. Stratified by sexual identity, separate multivariable models were fit for each outcome, using the same exposure variables.

Utilization of HIV Prevention Services (Table 2)

**Table 2:** Utilization of HIV prevention services.

N = 5971	CONDOMS			HIV TESTING		PrEP
	*AOR*	*95% CI*	*AOR*	*95% CI*	*AOR*	*95% CI*
**GAY MEN**						
**Criminalization**	1.43	0.94–2.18	**1.40**	**1.02–1.91**	0.62	0.24–1.61
**Sexual Stigma**	1.01	0.85–1.20	0.97	0.86–1.08	*0*.*78*	*0*.*60*–*1*.*02*
**Provider-Discrimination**	1.14	0.95–1.36	**1.49**	**1.25–1.78**	1.06	0.77–1.45
**Community Engagement**	**1.53**	**1.23–1.91**	**1.21**	**1.00–1.47**	**1.47**	**1.09–1.97**
**LGBT-Based Services**	**2.89**	**2.28–3.65**	**3.30**	**2.36–4.60**	**5.26**	**2.50–11.05**
**Psychological Wellbeing**	1.01	1.01–1.01	1.01	1.01–1.01	1.00	0.99–1.00
Men reporting use last 6 mos:	n = 3,574		n = 2,934		n = 394* / N = 3,107	
**BISEXUAL & OTHER MSM**						
**Criminalization**	0.74	0.41–1.32	0.99	0.57–1.70	2.12	0.67–6.69
**Sexual Stigma**	0.99	0.76–1.30	0.90	0.73–1.12	0.80	0.40–1.60
**Provider-Discrimination**	**1.58**	**1.10–2.28**	***1*.*38***	***0*.*94*–*2*.*02***	**2.14**	**1.18–3.89**
**Community Engagement**	1.74	0.85–3.56	**1.97**	**1.08–3.59**	1.40	0.92–2.14
**LGBT-Based Services**	**2.51**	**1.65–3.82**	**3.66**	**1.97–6.81**	**7.12**	**3.16–16.04**
**Psychological Wellbeing**	1.01	1.00–1.02	1.01	1.01–1.02	1.00	0.99–1.01
Men reporting use last 6 mos:	n = 971		n = 733		n = 90* / N = 894	

*Reflects having *ever* used PrEP; Bold reflects AORs with *p*<0.05; Italicized reflects AORs with *p*<0.10; Models adjusted for racial/ethnic minority status, participant age, insurance, ability to make ends meet, and country-level income.

**Among gay men**, utilization of *condoms* was associated with higher odds of community engagement (AOR = 1.53, [1.23–1.92], *p*<0.001), and receiving services from a LGBT-led organization (AOR = 2.89, [2.28–3.65], *p*<0.001). Utilization of *HIV testing* was associated with higher odds of living in criminalizing country (AOR = 1.40, [1.02–1.91], *p* = 0.04), health provider-discrimination (AOR = 1.49, [1.25–1.78], *p* = 0.001), community engagement (AOR = 1.21, [1.00–1.47], *p*<0.05), and receiving services from a LGBT-led organization (AOR = 3.30, [2.36–4.60], *p*<0.001). Utilization of *PrEP* was associated with lower odds of sexual stigma (AOR = 0.78, [0.60–1.02], *p* = 0.07); and higher odds of community engagement (AOR = 1.47, [1.09–1.97], *p*<0.01), and receiving services from a LGBT-led organization (AOR = 5.26, [2.50–11.05], *p*<0.001).

**Among bisexual and other MSM**, utilization of *condoms* was associated with higher odds of health provider-discrimination (AOR = 1.58, [1.10–2.28], *p* = 0.01), and receiving services from a LGBT-led organization (AOR = 2.51, [1.68–3.82], *p*<0.001). Utilization of *HIV testing* was associated with higher odds of health provider-discrimination (AOR = 1.38, [.94–12.02], *p* = 0.10), community engagement (AOR = 1.97, [1.08–3.59], *p*<0.03), and receiving services from a LGBT-led organization (AOR = 3.66, [1.97–6.81], *p*<0.001). Utilization of *PrEP* was associated with higher odds of health provider-discrimination (AOR = 2.14, [1.18–3.89], *p* = 0.01), and receiving services from a LGBT-led organization (AOR = 7.12, [3.16–16.04], *p*<0.001).

Utilization of HIV Care Services (Table 3)

**Table 3:** Utilization of HIV care services among men living with HIV.

	HIV CARE		ART	
	*AOR*	*95% CI*	*AOR*	*95% CI*
**GAY MEN**				
**Criminalization**	0.53	0.23–1.24	0.96	0.42–0.10
**Sexual Stigma**	***0*.*63***	***0*.*39*–*1*.*02***	0.68	0.23–1.13
**Provider-Discrimination**	0.87	0.59–1.29	**3.28**	**1.50–2.60**
**Community Engagement**	**1.62**	**1.04–2.55**	0.94	0.35–0.16
**LGBT-Based Services**	1.18	0.73–1.89	***0*.*68***	***0*.*16*–*1*.*66***
**Psychological Wellbeing**	0.99	0.98–1.00	0.99	0.01–1.91
Men reporting use last 6 mos:	n = 758 / N = 872 (living with HIV)		n = 732 / N = 758 (in HIV care)	
**BISEXUAL & OTHER MSM**			Did not converge	
**Criminalization**	1.32	0.36–4.91		-
**Sexual Stigma**	***0*.*16***	***0*.*02***–***1*.*38***		-
**Provider-Discrimination**	0.51	0.09–2.80		**-**
**Community Engagement**	3.65	0.66–20.07		**-**
**LGBT-Based Services**	1.32	0.18–9.52		**-**
**Psychological Wellbeing**	0.99	0.96–1.01		-
Men reporting use last 6 mos:	n = 89 / N = 104 (living with HIV)		n = 88 / N = 89 (in HIV care)	

Bold reflects AORs with *p*<0.05; Italicized reflects AORs with *p*<0.10; Models adjusted for racial/ethnic minority status, participant age, insurance, ability to make ends meet, and country-level income.

**Among gay men living with HIV**, utilization of *HIV care services* was associated with lower odds of sexual stigma (AOR = 0.63, [0.39–1.02], *p* = 0.06), and higher odds of community engagement (AOR = 1.62, [1.04–2.55], *p* = 0.05); and utilization of *ART* was associated with higher odds of health provider-discrimination (AOR = 3.28, [1.50–2.60], p = 0.01), and lower odds of receiving services from a LGBT-led organization (AOR = .68, [.16–1.66], *p*<0.10).

**Among bisexual and other MSM living with HIV**, utilization of *HIV care services* was associated with lower odds of sexual stigma (AOR = 0.16, [0.02–1.38], *p* = 0.10), The model for utilization of *ART* did not converge due to small the small sample size of bisexual and other MSM in HIV care.

Utilization of Sexual Health and Support Services (Table 4)

**Table 4:** Utilization of sexual health and support services.

N = 5971	STI TESTING		STI TREATMENT		MENTAL HEALTH CARE		COMMUNITY-BASED COUNSELING	
	*AOR*	*95% CI*	*AOR*	*95% CI*	*AOR*	*95% CI*	*AOR*	*95% CI*
**GAY MEN**								
**Criminalization**	1.28	0.86–1.89	1.36	0.81–2.26	0.93	0.63–1.38	0.95	0.55–1.65
**Sexual Stigma**	**0.89**	**0.79**–**1.00**	**0.80**	**0.69–0.93**	**0.85**	**0.76–0.96**	**0.85**	**0.73–0.99**
**Provider-Discrimination**	**1.65**	**1.26–2.16**	**1.51**	**1.30–1.77**	1.03	0.86–1.23	**1.23**	**1.05–1.45**
**Community Engagement**	**1.21**	**1.03–1.41**	***1*.*21***	***0*.*99*–*1*.*49***	**2.19**	**1.86–2.57**	**4.01**	**3.01–5.34**
**LGBT-Based Services**	**3.61**	**2.74–4.75**	**1.65**	**1.17–2.31**	**1.41**	**1.17–1.70**	**2.63**	**1.72–4.01**
**Psychological Wellbeing**	1.01	1.00–1.01	1.00	1.00–1.01	0.98	0.98–0.98	1.00	1.00–1.01
Men reporting use last 6 mos:	n = 3,047		n = 1,231		n = 1,029		n = 688	
**BISEXUAL & OTHER MSM**								
**Criminalization**	1.19	0.71–1.99	0.98	0.62–1.55	1.10	0.58–2.08	1.07	0.62–1.83
**Sexual Stigma**	0.89	0.71–1.11	0.78	0.53–1.14	**0.76**	**0.60**–**0.95**	0.64	0.37–1.12
**Provider-Discrimination**	**1.58**	**1.11–2.24**	**1.49**	**1.15–1.92**	**1.32**	**1.07–1.64**	**1.49**	**1.14–1.93**
**Community Engagement**	**1.56**	**1.11–2.18**	**1.45**	**1.17–1.80**	**2.48**	**1.77–3.46**	**2.51**	**1.70–3.70**
**LGBT-Based Services**	**3.89**	**2.43–6.25**	**1.94**	**1.58–2.39**	**1.49**	**1.06–2.11**	**3.35**	**2.30–4.88**
**Psychological Wellbeing**	1.01	1.01–1.02	1.00	0.99–1.01	0.98	0.98–0.99	1.00	0.99–1.01
Men reporting use last 6 mos:	n = 730		n = 262		n = 239		n = 220	

Bold reflects AORs with *p*<0.05; Italicized reflects AORs with *p*<0.10; Models adjusted for racial/ethnic minority status, participant age, insurance, ability to make ends meet, and country-level income.

**Among gay men**, utilization of *STI testing* was associated with lower odds of sexual stigma (AOR = 0.89, [0.79–1.00], *p* = 0.05); and higher odds of health provider-discrimination (AOR = 1.65, [1.26–2.16], *p* <0.001), community engagement (AOR = 1.21, [1.03–1.41], *p* = 0.002), and receiving services from a LGBT-led organization (AOR = 3.61, [2.74–4.75], *p*<0.001). Utilization of *STI treatment* was associated with lower odds of sexual stigma (AOR = 0.80, [0.69–0.93], *p* = 0.001); and higher odds of health provider-discrimination (AOR = 1.51, [1.30–1.77], *p*<0.001), community engagement (AOR = 1.21, [.99–1.49], *p* = 0.06), and receiving services from a LGBT-led organization (AOR = 1.65, [1.17–2.31], *p*<0.001). Utilization of *mental health care* was associated with lower odds of sexual stigma (AOR = 0.85, [0.76–0.96], *p* = 0.01); and higher odds of community engagement (AOR = 2.19, [1.86–2.57], *p*<0.001), receiving services from a LGBT-led organization (AOR = 1.41, [1.17–1.70], *p*<0.001). Utilization of *community-based support or self-help groups or individual counseling* was associated with lower odds of sexual stigma (AOR = 0.85, [0.73–0.99], *p* = 0.021); and higher odds of health provider-discrimination (AOR = 1.23, [1.05–1.45], *p* = 0.01), community engagement (AOR = 4.01, [3.01–5.34], *p*<0.001), and receiving services from a LGBT-led organization (AOR = 2.63, [1.72–4.01], *p*<0.001).

**Among bisexual and other MSM**, utilization of *STI testing* was associated higher odds of health provider-discrimination (AOR = 1.59, [1.11–2.24], *p* = 0.01), community engagement (AOR = 1.56, [1.11–2.18], *p* = 0.01), and receiving services from a LGBT-led organization (AOR = 3.89, [2.43–6.25], *p*<0.001). Utilization of *STI treatment* was associated with higher odds of health provider-discrimination (AOR = 1.49, [1.15–1.92], *p*<0.001), community engagement (AOR = 1.45, [1.17–1.80], *p*<0.01), and receiving services from a LGBT-led organization (AOR = 1.94, [1.58–2.39], *p*<0.001). Utilization of *mental health care* was associated with lower odds of sexual stigma (AOR = 0.76, [0.60–0.95], *p* = 0.02); and higher odds of health provider-discrimination (AOR = 1.32, [1.07–1.64], *p* = 0.01), community engagement (AOR = 2.48, [1.77–3.46], *p*<0.001), and receiving services from a LGBT-led organization (AOR = 1.49, [1.06–2.11], *p* = 0.02). Utilization of *community-based support or self-help groups or individual counseling* was associated with higher odds of health provider-discrimination (AOR = 1.49, [1.14–1.93], *p*<0.01), community engagement (AOR = 2.51, [1.70–3.70], *p*<0.001), and receiving services from a LGBT-led organization (AOR = 3.35, [2.30–4.88], *p*<0.001).

## Discussion

Consistent with previous research [26, 27], our study findings show a drop off in HIC care and prevention services utilization, pointing to the importance of retaining gay and bisexual men in care across the continuum. Overall, as hypothesized, structural indicators of services utilization revealed a negative impact of sexual stigma on health care utilization. This conforms with prior research showing a link between sexual stigma and reduced health seeking behavior among gay and bisexual men [28], suggesting the necessity for structural level interventions to reduce sexual stigma.

Contrary to our prediction and research showing negative associations between provider-discrimination and services access [13, 26], provider-discrimination odds were higher among those utilizing many of the services. We posit that this may have resulted from greater opportunities to experience provider-discrimination by men who utilized services during the previous six months. Future research is needed to uncover how gay and bisexual men navigate provider-discrimination experiences they may encounter to attend to their health care needs; as well as examine the impact that provider discrimination may have on treatment adherence and outcomes. In addition, research should examine the specificity of health care provider-discrimination practices. Such data could inform the design of provider training interventions that can lead to appropriate services for gay and bisexual men.

Although bivariate analyses confirmed associations between ecological and individual level indicators, and utilization of services outcomes, multivariable analyses yielded only one significant positive association for gay men utilizing HIV testing. A possible explanation is that ecological and individual level factors are more salient when attempting to access services than for utilization of services. Additionally, the lack of association between criminalization and utilization of most services may be a result of the study’s predominantly urban and highly educated sample having skewed the results. Interestingly, demographic characteristics reveal that most gay and bisexual men were unable to adequately meet their financial needs despite their reporting high levels of education. This is not surprising given the structural violence, such as stigma and discrimination, they face on a regular basis [9, 25, 29–33].

As hypothesized, both community level indicators were positively associated with utilization of services utilization. Community engagement may serve to buffer stigma and discrimination impact. Possible mechanisms explaining the effect of gay and bisexual men gathering socially include the extent to which this fosters self-esteem, encourages health-promoting peer norms, and provides social support and a sense of belonging [34, 35]. In addition, apart from a negative association with ART utilization (which, we speculate, may reflect a lack of LGBT-led organizations with sufficient resources to offer ART), the highest odds of utilizing most services were found for those using services delivered by LGBT-led community-based services, reinforcing the importance of community-led HIV responses. Indeed, a recent review of the literature supported a comparative advantage of community-led direct services, including improved HIV-related knowledge, attitudes, intentions, self-efficacy, risk behaviors, risk appraisals, health literacy, treatment adherence, and viral suppression [36]. The authors recommended that prevention programs, especially those intended for people living with and disproportionately affected by HIV, be community-led [36].

While the pattern of utilization outcomes was similar for both gay and bisexual men, bisexual men had higher odds of reporting provider discrimination when utilizing condom programs, PrEP, and mental health services than gay men. In addition, they had higher odds of using LGBT-led organizations or programs when utilizing PrEP, and community-based support or self-help groups or individual counseling.

Our findings stand in contrast to previous research findings that, compared to gay men, bisexual men were significantly less likely to report multiple forms of discrimination including from domains such as health care, the public sphere, and employment and training [37]. In a study of psychological well-being, bisexual people reported a lack of connectedness to the LGBT community [38]. However, the same study also found the association between social well-being and bisexuality was fully mediated by community connectedness. It is rare to find studies that can disaggregate between the two groups. More research is needed that explores similarities and differences in sexual health and health utilization between gay and bisexual men.

Study findings must be qualified by several limitations. First, survey translations may have contributed to poor construct validity in some languages, in turn affecting their scale reliability. Future analyses by language are warranted to assess this possibility. Second, survey data were gathered using a convenience sample, creating possible selection bias for gay and bisexual men who are socially connected to HIV or LGBT organizations or online LGBT communication infrastructure, as well as for those who have Web and e-mail access, limiting participation of gay and bisexual men in regions where internet access may be restricted. This may have led to overestimating utilization of services and community engagement, and underestimating structural violence. Further, the study sample was disproportionately urban and highly educated, which may account for non-significant associations between criminalization and utilization of health services. Importantly, this and the use of a nonprobability internet sample that overrepresented Latin America and the Caribbean prohibits generalizability to all gay and bisexual men. Third, the use of a cross-sectional study design prohibits attribution of causality between structural or community level factors and utilization of services. Fourth, the sample size of bisexual men in this study prevented us from examining associations for utilization of ART among bisexual men. Finally, our analyses did not account for multiple testing, given the exploratory nature of our analyses. Hence, it is possible that some of our findings may be due to type 1 error. However, because the associations we observed are broadly consistent with the literature and our understanding of the structural barriers and facilitators of utilization of services, we have greater confidence in the validity of our results [8–12, 25, 27, 32, 33, 39–41].

## Conclusions

This study contributes to a growing body of evidence calling for efforts to ensure both access to and utilization of basic HIV prevention, HIV-care, and support services for gay and bisexual men [42]. Beyond addressing criminalization at the country level, barriers to and enablers of health services utilization must be addressed at structural, community and individual levels. Specifically, findings support the need for structural interventions designed to reduce sexual stigma, train and sensitize healthcare providers, and support community level strategies that bring gay men together to support each other and lead service provision engagement. Location of services and who delivers them also matter, highlighting the importance of LGBT-led and community-based services, a conclusion supported by previous research focused on the comparative advantage of community- or peer-led services [43–45]. Finally, stigma and discrimination in any form are inherently problematic from the perspective of public health and human rights. Securing the health and human rights of gay and bisexual men, efforts to combat sexual stigma, and improving provision of services are essential to HIV prevention and care strategies to change the trajectory of the HIV pandemic among gay and bisexual men.

## Supporting information

S1 Data (XLSX)

S2 Data (DTA)
